# The Bending Properties of Hybrid Cross-Laminated Timber (CLT) Using Various Species Combinations

**DOI:** 10.3390/ma16227153

**Published:** 2023-11-14

**Authors:** Ahmed Altaher Omer Ahmed, József Garab, Erika Horváth-Szováti, János Kozelka, László Bejó

**Affiliations:** 1 Institute of Wood Technology and Technical Sciences, University of Sopron, 9400 Sopron, Hungarybejo.laszlo@uni-sopron.hu (L.B.); 2 Institute of Informatics and Mathematics, University of Sopron, 9400 Sopron, Hungary; 3 Faculty of Wood Engineering and Creative Industries, University of Sopron, 9400 Sopron, Hungary

**Keywords:** cross-laminated timber, hardwood, bending strength, modulus of elasticity, polyurethane resin

## Abstract

Cross-laminated timber (CLT) has become a massive commercial success in recent years due to its high performance, technological advantages, and low environmental impact. The finite softwood raw material supply has motivated researchers to find alternatives. This study presents an investigation of the viability of some Hungarian hardwood materials, such as CLT materials. Homogeneous beech, poplar, and spruce panels, as well as their combinations, were created using a polyurethane adhesive. The experimental results show the clear potential of Hungarian poplar, which performed much better than spruce. Poplar’s modulus of elasticity (MOE) and modulus of rupture (MOR) values reached or exceeded those of high-grade commercial softwood CLT. The bending properties of beech and hybrid beech–poplar panels far exceeded the performance of commercial panels, which shows the excellent potential of high-density hardwoods for high-performance CLT production. Beech–spruce hybrid panels seriously underperformed. This was caused by gluing issues, probably due to the large density differences between the two species, as evidenced by the glueline failure exhibited by most of these specimens during testing. The average panel density proved to be the best predictor of mechanical performance, except for beech–spruce hybrid panels.

## 1. Introduction

In the past two decades, modern mass timber construction went from a relative novelty to a widely accepted and used form of construction, with an estimated annual volume of 2.8 million m^3^ of CLT in 2020 [1]. A variety of factors contributed to its rapid growth, including environmental issues, like the depletion of required non-renewable resources and high emissions related to concrete construction, and technological factors, like fast and dry construction, low weight, and the excellent specific mechanical properties of CLT, among others.

The concept of CLT was first developed in Germany and Austria in the 1990s, and it was based on various softwood species used as raw materials [2]. As with any other type of wood-based construction, softwood is eminently suited for mass timber products: Its regular growth characteristics, even grain structure, and reliable mechanical performance, among other factors, make it a natural raw material for CLT production. In the meantime, there is an increasing concern that raw material resources will not be able to keep pace with the ever-increasing demand for mass timber. Research has intensified with respect to finding alternative raw materials, including various hardwood species.

Hungarian forests consist of 85% hardwood and 15% softwood timberland [3]. Consequently, there is a rich tradition of hardwood utilization and research, including research on the use of hardwood for construction. This includes a small-scale study that created CLT out of some Hungarian poplar (*Populous* × *euramerica* cv. I-214) raw materials, with promising but inconclusive results [4]. This study aims to conduct more in-depth research using multiple species, combined and single-species panels, and larger sample sizes. The study focuses primarily on bending properties.

CLT manufactured from hardwood offers opportunities for taking advantage of underutilized or low-value hardwoods in order to provide new applications. Among others, oak, beech, and ash have higher densities and greater strength than spruce or pine. This makes them ideal for use in structural applications [5]. Manufacturing hybrid CLT may be the best way to realize the benefits of using various wood species. As experimental and theoretical results have revealed, beech and hybrid poplar–beech CLT have tremendous potential for structural performance in the construction and building sector. Hardwood, with its good mechanical properties, may be the key to creating long-span and high-stress timber constructions [6,7,8].

The manufacturing process of hardwood CLT is similar to softwood; the wood is first cut into boards and then glued together in layers. However, the denser nature of hardwoods can make them more difficult to machine and may require adjustments to the manufacturing process. Therefore, there are some challenges associated with using hardwood in CLT production. Additionally, hardwood tends to be more prone to cracking and splitting, which could lead to defects in the final product. Manufacturers have highlighted that the first step to the successful implementation of hardwood or hardwood–softwood hybrid CLT would require the production of dimensional-grade hardwood lumber via hardwood sawmills. However, manufacturing CLT is achievable, and the resulting product offers interesting perspectives for specific applications [9,10].

Scientific attention in the last decade has very actively turned to hardwood species as raw materials for CLT manufacturing purposes. The results of Zöllig [11] showed that hardwood CLT made from beech or ash has good potential for construction purposes based on its mechanical properties. Furthermore, the thickness of the lamella is of great importance for the panel’s performance. Several researchers in the USA investigated an underutilized, low-grade hardwood: yellow poplar. Hovanec’s results [12] showed that laminate orientation and adherend thickness affected bond strengths. However, no differences were observed with respect to failure or delamination when compared with low-grade softwood CLT. Mohamadzadeh and Hindman [13] conducted a study on yellow poplar CLT based on the relevant APA standard [14]. The results of the tests showed that the bending stiffness, bending strength, and resistance to delamination surpassed the minimum requirements in the standard. Interestingly, the shear strength of yellow poplar CLT exceeded that of softwood species, such as hard pine, by more than 20%.

According to Torno and van de Kuilen [15], the high costs associated with hardwood components pose challenges to the market success of CLT panels. They investigated CLT panels made using beech and ash components. The authors put forth a hypothesis suggesting that the suboptimal processes involved in converting hardwood logs into CLT components contribute to these high costs. The findings of the study revealed that the yield, expressed as a percentage, increases as the log diameter grows.

Several researchers assessed the suitability of poplar and hybrid poplar as potential raw materials for CLT production. Environmentally certified (FSC) hybrid poplar (*Pacificus albus*) of low density (specific gravity of 0.35) was used to produce and test ten CLT panels according to the North American ANSI/APA PRG 320 standard. According to the authors, hybrid poplar CLT demonstrated that its shear and bending strength values surpassed the requirements set by the standard. However, the modulus of elasticity (MOE) of hybrid poplar CLT was found to be lower than the E3 grade specified in the standard [16]. Meanwhile, poplar received attention in Europe as well. The technical feasibility of CLT panels made of Italian hybrid poplar (*Populus × euroamericana*) and chestnut was demonstrated when panels were manufactured and tested according to European standard EN 789 [17,18].

Hungary has the second largest poplar plantation in Europe after France, with 200-thousand hectares in area [19]. There has been substantial scientific interest in utilizing this species for creating construction materials, including a preliminary investigation that is directly connected to our study. Technical feasibility was demonstrated in Hungary with respect to using poplar lumber to create CLT. The MOR values of poplar CLT were comparable to those of softwood panels, while unfortunately, the MOE values fell short. Further investigation is necessary to establish the economic viability and technological conditions for the commercial production of poplar CLT [4]. Our current study complements these findings with further measurement results.

Due to promising experimental results, several architectural projects emerged during the last decade. One example is the Endless Stair project, presented at the London Design Festival in 2013; yellow poplar CLT panels were used [20]. Beech CLT was used for building a new factory and the headquarters of the furniture firm Vitsoe in 2017 [21]. Moreover, the Austrian forest products firm Haaslacher Norica already started producing commercially available birch CLT, which served as the raw material for a house built as a pilot project [22].

Although hardwood materials have some drawbacks due to their growth characteristics, wood-based construction materials offer opportunities for value-added utilization, such as glued structural materials or wood-based structural composites [23]. Therefore, using hardwood for CLT presents opportunities for sustainable forest management, economic development in rural communities, and the utilization of underutilized hardwood resources. In order to investigate the properties of hardwood CLT further, Hungarian hardwood raw materials were the focus of this study.

## 2. Materials and Methods

The presented study involved three species: two hardwood and a softwood species. European beech (*Fagus sylvatica*) is a relatively high-density species with good mechanical properties. It is widespread in Hungary, and it is used for many purposes. Poplar wood (*Populus* spp.) is one of the most widespread species in Hungary and includes several species and natural hybrids. It has low density and moderate load-bearing capacities, similarly to lightweight softwoods [3]. Norway spruce (*Picea abies*) is one of the standard species used for CLT production, and it was included for reference and to create softwood/hardwood hybrid panels. Cross-laminated timber specimens consisted of various combinations of these three species. In the case of poplar, a commercially available material was used. Whether it was the exact species or a clone was impossible to determine. It was most likely a natural hybrid variety. The raw material characteristics are as follows:

- Dried C24-grade Norway spruce (dim: 6000 × 150 × 50 mm) with an average moisture content (MC) of 10%;
- Commercial-grade dried beech (dim: 2000 × 150 × 45 mm) with an average MC of 7%;
- Green poplar lumber, ungraded (dim: 2000 × 130 × 40 mm).

Green poplar lumber was dried to a target MC of 12% (actual MC between 10 and 12%) using a small laboratory convection drier. All raw materials (regardless of prior grading) were stress-graded according to MSZ EN 338 [24] using the PLG + lumber grader equipment developed at the Jozsef Bodig Wood NDT Laboratory of the University of Sopron [25]. The outer layer consisted of higher grades (typically at least C24 or D50), while lower grades were used as core lamellas.

**Figure 1:**
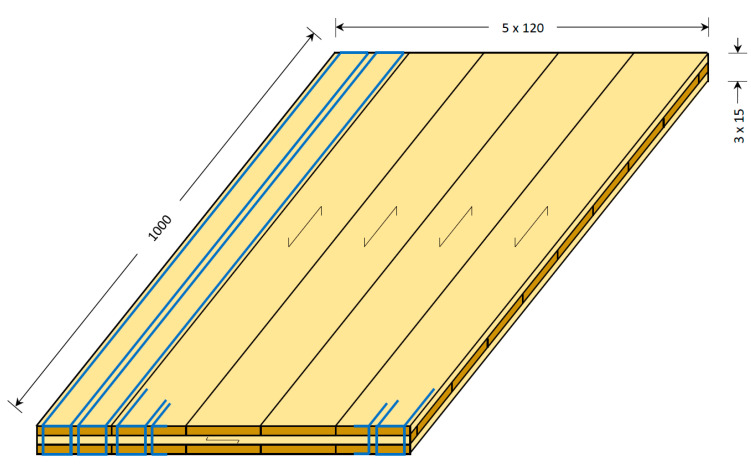
Experimental CLT panels. Blue outlines show the position of the bending specimens. Dimensions are in mm.

**Figure 2:**
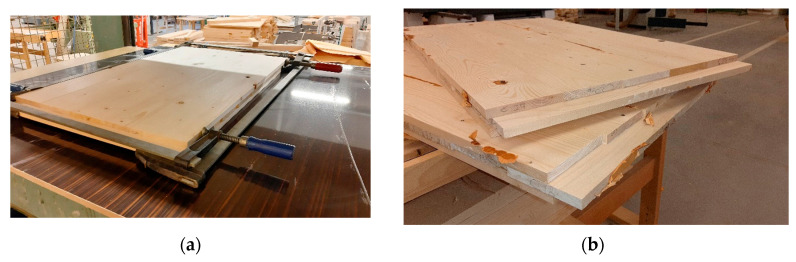
Manufacturing CLT: (**a**) glue application; (**b**) and the completed layup.

Six 3-layer CLT panels, 1000 × 600 × 45 mm in size, were manufactured using various species combinations, as shown in Table 1. The adhesive used comprised the Jowapur^®^ 681.60 (Jowat Swiss AG, Buchrainon, Switzerland) liquid one-component polyurethane adhesive graded for load-bearing structural wood bonding. CLT manufacturing and specimen preparation followed the protocol outlined below:

- Preparing planed lamellas sized 1000 × 120 × 15 mm and 600 × 120 × 15 mm for longitudinal and crossband lamellas, respectively
- Wetting the surface of beech lamellas to increase the surface MC to approx. 9% in order to decrease MC differences between layers and provide the necessary moisture for polyurethane resin curing;
- Creating the layout for the bottom, middle, and top layers from the appropriate lamellas, as shown in Table 1 (one panel each). The top and bottom layers consisted of 5 lamellas, while 9 crossband lamellas made up the core (see Figure 1). Two layups were created simultaneously;
- Applying the adhesive to the bottom and middle layers, using spackle knives to spread the resin on the surface of the layup. The adhesive quantity was 200 g/m^2^, according to the highest value recommended by the manufacturer;
- Installing screw clamps at the end of each layer (Figure 2a) to eliminate gaps between the lamellas as much as possible (lamellas were not side-glued);
- Applying a pressure of 0.8 N/mm^2^ to the panel, using a 2 × 1.2 m hydraulic veneering press (pressing two panels side by side) for the duration of 3 h. Before opening the press, adhesive curing was verified by checking the material squeezed out;
- Removing the panels from the press and setting them aside for several days for complete curing (Figure 2b).

The above procedure was repeated 3 times for a total of 6 panels according to the combinations shown in Table 1. After complete curing, the panels were cut into bending specimens sized 1000 × 45 × 45 mm using a circular table saw. Each of the face layers consisted of 5 lamellas side by side, and 2 specimens were cut from each face lamella region (see Figure 1) for a total of 10 specimens per panel. Specimens cut from different face lamellas can be regarded as independent of each other, except for the common middle lamella, which has little effect on the longitudinal bending properties of CLT.

**Figure 3:**
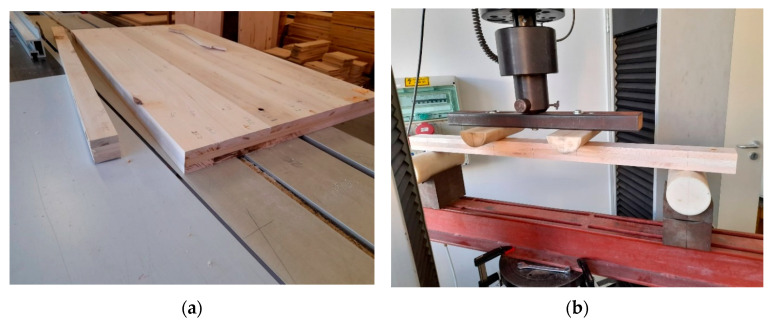
Test samples and test setup: (**a**) cutting the bending specimens; (**b**) the 4-point bending test.

A total of 60 CLT specimens were tested with Instron 4208 universal testing machine (Instron Coorporation, Norwood, MA, USA) in a 4-point bending procedure according to MSZ EN 408_2010+A1_2012 (global bending MOE method) [26], following standard specifications in terms of testing parameters and using a loading rate of 8 mm/min (Figure 3). The spruce and poplar G modulus values required for the MOE calculations were based on values from the literature [27], and they were determined using dynamic torsion vibration measurements in the case of beech. After the completion of the test, the failure mode of the specimens was also documented. Most specimens exhibited a combination of several failure modes, all of which were recorded, starting with the most dominant one. Moisture content and density specimens were cut from the end of each specimen, measured for dimensions and weight, and dried at 103 ± 2 °C to determine the MC and density.

Statistical data analysis included ANOVA to determine between-panel, within-panel, and within-lamella variations, and regression analysis was carried out to compare the results to the stress grade of the face lamellas and the density of the panels.

## 3. Results and Discussions

**Figure 4:**
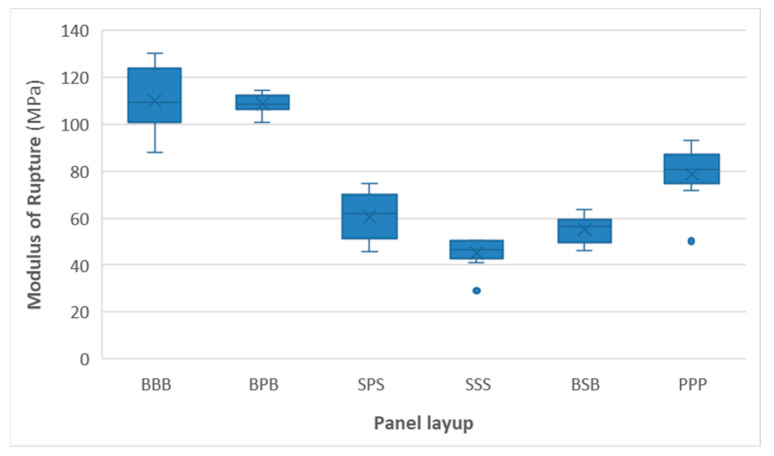
The bending strength of various types of panels. Blue dots are outlier values.

**Figure 5:**
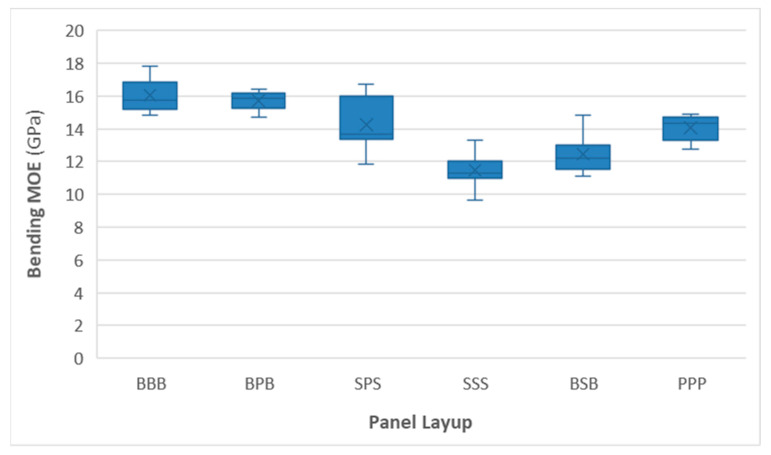
The bending MOE of various types of panels.

Figure 4 and Figure 5 show the distribution of measured MOR and MOE values for all panels. Remarkably, panels containing spruce layers have the lowest mechanical performance, with the uniform spruce (SSS) panel having the lowest MOR and MOE values. Full poplar panels had somewhat higher strength and stiffness, even though their face lamella grades were typically lower (C22 to C27) compared to those of spruce (C24 to C35). As expected, panels with beech as the face layer (BBB and BPB) had the highest MOR and MOE values (beech-face lamellas were typically graded as D40 or D50, with occasional D60 grades). Differences in MOR were more pronounced than those in MOE.

The nested design ANOVA results (Table 2) revealed that the effect of CLT composition (wood species used in the panel) is highly significant, while the effect of lamellas within those panels did not significantly affect the MOR of the specimens. Based on Tukey’s HSD test, most panels were significantly different from one another, except in the case of SSS vs. BSB and BBB vs. BPB. ANOVA results were similar to MOE.

**Figure 6:**
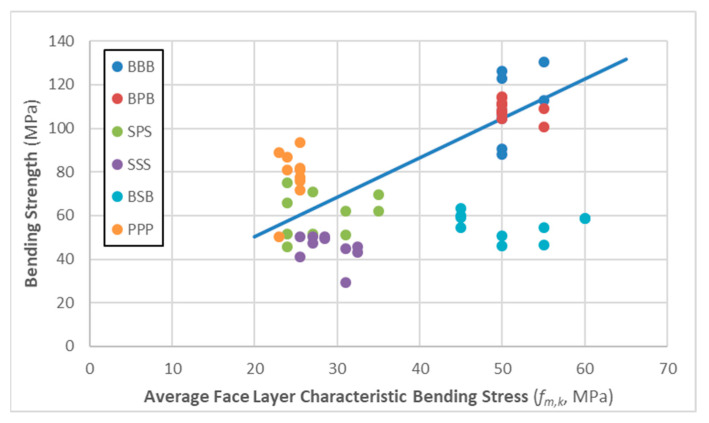
The relationship between average face layer characteristic bending stress and the measured bending strength values across the various panel types. Note: BSB data points were excluded when creating the trendline.

The diagram in Figure 6 compares the average face-layer characteristic bending design stress (*fm,k*, based on MSZ EN 338) to the bending strength of panels. If we exclude BSB specimens, which seriously underperformed compared to other panels, there is a more-or-less linear relationship between the two parameters (see trend line shown in graph). Even so, homogeneous spruce panels still underperformed compared to the face-layer grades, while homogeneous poplar specimens exceeded expectations.

**Figure 7:**
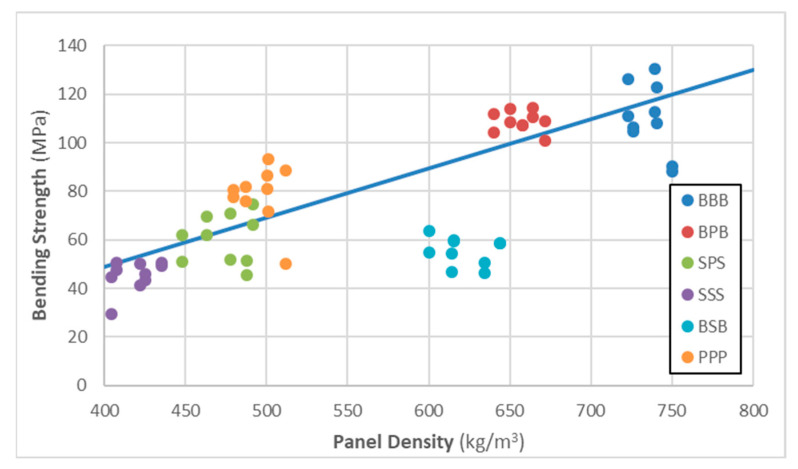
The relationship between the measured panel density and the measured bending strength values across various panel types. Note: BSB data points were excluded when creating the trendline.

Figure 7 shows the relationship between measured panel density and MOR. Based on this graph, there is a strong linear relationship between density and bending strength. This provides a possible explanation for the somewhat unexpected underperformance of the spruce panels, which had the lowest density of the measured panels. Since most of the lamellas were homogeneous, straight-grained materials without large knots or other significant defects, density is likely to be a strong predictor of the mechanical performance of the lamellas and therefore the CLT panels. Since wood is a naturally variable material, there are other influencing factors as well, including glueline strength, which caused hybrid beech–spruce specimens to have relatively low bending strength values compared to their density.

BSB specimens are outliers in this respect, performing poorly compared to their density. Failure mode analysis reveals a probable explanation for this. Various specimens showed a variety of failure modes, and failure was often a combination of several modes. Rolling shear was the dominant failure mode in homogeneous poplar and spruce panels, as well as in BPB panels. Homogeneous beech and SPS panels exhibited a wide variety of failure modes, but the most typical failure was tensile or brittle–tensile failure at the tension side of the specimens.

**Figure 8:**
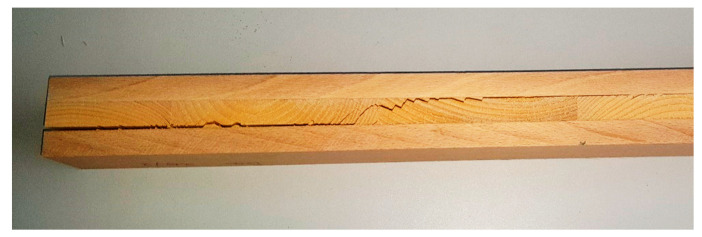
Partially delaminated BSB specimen.

In contrast, most BSB specimens failed primarily via glueline delamination (Figure 8), and this occurred probably long before reaching the ultimate tensile or compression stress of the face lamellas or the shear strength of the core. The exact reason for this is unknown, but it is most likely a result of the excessive difference (more than 70%) in the density of the two species. Based on observations, such large differences often lead to weaker gluelines, which may affect both the deformation and the ultimate strength/failure mode of the glued structural member. Notably, BSB MOE is also somewhat low compared to the density, but the difference is not as pronounced as that shown in Figure 7.

The experimental results were compared to the properties of commercially available softwood products based on their technical data sheets (Table 3). In terms of mechanical parameters, all experimental panels fulfilled the requirements of lower-grade panels (MOE of 11,000–14,700 MPa and an MOR of 16–24 MPa). Both low-density experimental and commercial softwood panels show some variation, but the stiffness of the experimental boards was generally within the range of commercial panels, while their bending strength (calculated as the fifth percentile of the experimental values) typically exceeded them, especially in the case of homogeneous poplar CLT. The homogeneous spruce panel was close to the lowest commercial grades in terms of MOE, while the rest of the panels all exceeded these values.

Table 4 and Table 5 represent the measured mechanical parameters of experimental CLTs, including the bending modulus of elasticity (MOE) and bending strength (MOR), along with some reference values for CLT panels made of other wood species with similar densities. Table 4 shows low-density panels made of softwood and poplar, and Table 5 includes panels containing beech layers.

The experimental results show that the CLT with uniform wood species exhibited the following increasing order based on the mean of the two measured mechanical properties:

(1) Spruce (MOE = 11,480 MPa and MOR = 45.19 MPa);
(2) Poplar (MOE = 14,070 MPa and MOR = 78.72 MPa);
(3) Beech (MOE = 16,040 MPa and MOR = 110.10 MPa).

Earlier studies indicated that poplar CLT panels may be viable. Although the shear and bending performance were acceptable, some studies show that the bending stiffness is within the lower region. Marko et al. observed 7898 MPa [4], Kramer et al. measured 7360 MPa [16], Vetsch found a value of 8068 MPa [36], and Das et al. measured 7907–8183 MPa during bending [33]. Our current results present a higher MOE (14,700 MPa). The strength class of lamellas was determined using a machine in the study reported by Marko et al. [4], which only discussed poplar CLT. The classes of face lamellas varied from C18 to C27. However, the strength class of the face lamellas in the present study varied from C22 to C27 in the case of uniform poplar panels. Therefore, the raw material in the current study had better mechanical properties. Furthermore, the density of the poplar wood used in this study was 496 kg/m^3^. The density of the raw material in the studies of Kramer et al. [16] and Das et al. [33] were significantly lower: 347 kg/m^3^ and 385 kg/m^3^, respectively. Although the density of the aspen in the work of Vetsch [36] was 500 kg/m^3^, difficulties arose with respect to the clamping of the panel during drying. He concluded that improving the clamping arrangement using roughly sanded wood and increasing the sample size would probably lead to outcomes that are more favorable. Our study shows that higher density and higher strength classes lead to better elastic properties in the case of uniform poplar panels.

Our beech results are comparable with the reference values obtained from the literature. Table 5 shows that both the MOE and MOR exceed the presented values in the study reported by Franke [6]. The difference between the results may be because Franke applied higher spans on larger CLT panels, in addition to more layers.

Using different wood species, the core layer influences the mechanical properties in a positive way. The apparent MOE and MOR increased when beech wood was used as the middle layer in a three-layer poplar–beech hybrid CLT [8]. The feasibility study of Wang et al. [34] demonstrated that softwood–poplar CLT panels were similar to those made of uniform softwood. Our study shows that benefits can be achieved when hybrid layups are applied with poplar. In the case of SSS panels, if the core layer is changed to poplar, the mechanical properties increase (MOE from 11,480 MPa to 14,360 MPa and MOR from 45.19 MPa to 60.51 MPa). In the case of BBB panels, if the face layers are changed to poplar, the mechanical properties do not decrease significantly (MOE from 16,040 to 15,700 MPa and MOR 110.10 to 108.73 MPa). Therefore, poplar has potential application as both a face layer and core layer in the case of hybrid layups.

In the case of hybrid wood species layups, the increasing order based on the mean of the two measured mechanical properties is as follows:

(1) Beech–spruce–beech (MOE = 12,450 MPa and MOR = 55.23 MPa);
(2) Spruce–poplar–spruce (MOE = 14,260 MPa and MOR = 60.51 MPa);
(3) Beech–poplar–beech (MOE = 15,700 MPa and MOR = 108.73 MPa).

Generally, the high-strength beech face layer improved the mechanical properties of CLT. The glueline delamination of BSB samples shows that the glueline’s strength was likely insufficient; therefore, the measured values are much lower than expected. Besides the difference between the densities of the two species, another explanation of the delamination issue may be that delamination occurs when a material undergoes various stresses that are primarily caused by variations in swelling and shrinkage, leading to drying defects [37]. Furthermore, the structural bonding of beech wood is a concern for the effective production and safe use of CLT. Brunetti et al. [38] investigated the bonding quality of beech–spruce–beech CLT with respect to three adhesives (one-component polyurethane—PUR; PUR + primer; and melamine–urea–formaldehyde—MUF). None of the tested adhesives met the requirements of delamination tests provided by the current standard for softwoods. The adhesive used in our study was one-component polyurethane, which may have also contributed to the lower mechanical performance of beech–spruce–beech samples. Furthermore, the research of Li et al. [39] demonstrated that bond directions show significant positive and negative effects when Radiata Pine (*Pinus radiate D. Don*) and Shining Gum (*Eucalyptus nitens*) hybrids were used in engineered timber products. Hence, it is advisable to consider the bond’s orientation when designing hybrid cross-laminated timber.

In the meantime, bonding issues did not seem to affect homogeneous beech and beech–poplar hybrid experimental CLT. Based on the excellent mechanical performance of beech wood, if proper bonding can be achieved (as indeed it was in the case of BBB and BPB panels), beech can be a promising CLT raw material candidate. Our experimental results confirmed that poplar is also a feasible solution for CLT if high-quality raw material is used, and proper bonding is attained. Summary statistics of the measured mechanical and physical properties of the various experimental CLT materials and experimental data for each CLT specimen are available in the supplementary materials (Tables S1 and S2).

Focusing on locally grown poplars may be a good opportunity for the value-added utilization of Hungarian hardwoods. The investigations can be extended to other drought-tolerant wood species as well, like Turkey oak.

## 4. Conclusions

The experimental investigation of Hungarian poplar and beech as a possible raw material for hardwood CLT, based on the bending properties of the created panels, revealed the following insights:

- The best predictor of the mechanical performance of the panels was overall panel density rather than the face layer’s strength class. Hybrid beech and spruce panels did not follow this trend, showing substantially worse performance compared to their average density due to inadequate bonding between the layers.
- Spruce panels used as controls in the experiment showed relatively low performance, corresponding to low-grade commercial CLT. This is most likely due to the low density of the raw material used in the experiment.
- Homogeneous poplar panels performed as well as or better than commercially available European softwood CLT panels. This is based on relatively high-density raw poplar materials, and this shows that high-quality poplar may be a viable alternative for CLT production; producing CLT may be a good opportunity for the value-added utilization of poplar.
- Homogeneous beech, as well as hybrid beech–poplar CLT, far outperformed even high-grade commercial CLT, especially in terms of the MOR. This indicates the excellent potential of high-density hardwood species in creating high-performance construction panels.
- Experimental hybrid beech–spruce CLT panels seriously underperformed compared to its high-density and high-strength face layers. This is due to insufficient glueline strength, which is most likely caused by the large density differences between the two species, combined with the general gluing issues of beech.

Further research is required to establish the additional mechanical and physical characteristics of hardwood and hybrid CLT. In the case of hybrid CLT panels, in particular, the assessment of shear stresses seems to be relevant. Shear test results would probably reveal the diversity of the selected wood species compared to bending tests.

## Figures and Tables

**Table 1:** Species combinations used in CLT experiments.

	Orientation	BBB	BPB	SPS	SSS	BSB	PPP
Top Layer	Longitudinal	Beech	Beech	Spruce	Spruce	Beech	Poplar
Middle Layer	Crossband	Beech	Poplar	Poplar	Spruce	Spruce	Poplar
Bottom Layer	Longitudinal	Beech	Beech	Spruce	Spruce	Beech	Poplar

**Table 2:** Nested design ANOVA table of the measured MOR values.

Source	SS	doF	MS	F	p
Intercept	2752	1	2752	30.59	0.000
Panel	23,271	5	4654	51.72	0.000
Lamella	1.74	1	1.74	0.019	0.890
Error	4769	53	89.98		

**Table 3:** Mechanical properties of SSS experimental results and some commercially available softwood CLT. The load is perpendicular to the CLT.

Panels	Lamella Strength Classes	MOE (MPa)	MOR ^1^ (MPa)
BBB	D40 to D60	16,040	89.2
BPB	face: D40 to D60 core: C18 to C24	15,700	102.4
SPS	face: C24 to C35 core: C18 to C24	14,260	48.1
SSS	face: C24 to C35 core: C20 to C24	11,480	34.5
BSB	face: D40 to D60 core: C20 to C24	12,450	46.4
PPP	face: C22 to C27 core: C18 to C24	14,070	59.9
MM-Holz 1 [28]	C24/T14	11,600	24.6
MM-Holz 2 [28]	C30/T18	12,600	30.7
Hasslacher 1 [29]	CL26E11.8	11,800	24.6
Hasslacher 2 [29]	CL36E14.7	14,700	33.5
Pfeifer [30]	Face layer: C24 Core layer: C16 ≤ 10%; C24 ≥ 90%	11,000	24.0
KLH [31]	C16 ≤ 10%; C24 ≥ 90%	12,000	24.0
Stora Enso 1 [32]	C16/T11	8000	16.4
Stora Enso 2 [32]	C24/T14	12,000	24.6

^1^ Fifth percentile value of experimental panels vs. declared characteristic values of commercial products.

**Table 4:** Comparison of the average mechanical properties of low-density experimental CLT with similar hardwood CLT from earlier studies.

Mechanical Properties	SSS	SPS	PPP	Homogeneous Poplar [16]	Homogeneous Aspen [33]	D.fir- Poplar- D. fir [34]
MOE (MPa)	11,480	14,260	14,070	7360	7907 to 8183	8070
MOR (MPa)	45.19	60.51	78.72	26	30.35 to 31.29	31.56

**Table 5:** Comparison of the average mechanical properties of experimental beech CLT with similar experimental CLT from earlier studies.

Mechanical Properties	BBB	BPB	BSB	Homogeneous Beech [6]	Spruce-Beech-Spruce [35]	Poplar-Beech-Poplar [8]
MOE (MPa)	16,040	15,700	12,450	12,306	10,400	880
MOR (MPa)	110.10	108.73	55.23	43.8	46.6	12.2

## Data Availability

Experimental data for individual specimens are available upon request from László Bejó at bejo.laszlo@uni-sopron.hu.

## Supplementary Materials

The following supporting information can be downloaded at https://www.mdpi.com/article/10.3390/ma16227153/s1. Table S1: Summary statistics of the measured mechanical and physical parameters of the experimental panels. Table S2: Experimental data for each CLT specimen.
