# Supplementary material for: The Bending Properties of Hybrid Cross-Laminated Timber (CLT) Using Various Species Combinations
Source: Materials (Basel). 2023 Nov 14;16(22):7153. doi: 10.3390/ma16227153 (PMC10672904; doi:10.3390/ma16227153)
Supplement: Supplementary file 1 [file materials-16-07153-s001.zip › materials-2686989-supplementary.pdf]

Table S1. Summary statistics of the measured mechanical and physical properties of the various experimental CLT materials (n=10)

**Layup**		**MOR** (MPa)	**MOE** (GPa)	**MC**	ρ (kg/m^3)
BBB	***Mean***	***110.10***	***16.04***	***6.94%***	***735.9***
	STD	14.00	1.05	0.03%	10.6
	Max.	88.22	14.81	6.90%	723.0
	Min.	130.50	17.80	6.98%	750.1
BPB	***Mean***	***108.73***	***15.70***	***6.98%***	***656.9***
	STD	4.21	0.58	0.13%	11.6
	Max.	100.82	14.68	6.84%	639.9
	Min.	114.42	16.40	7.20%	671.8
SPS	***Mean***	***60.51***	***14.26***	***7.35%***	***473.50***
	STD	10.01	1.55	0.12%	16.9
	Max.	45.60	11.83	7.16%	448.0
	Min.	74.84	16.71	7.48%	491.2
SSS	***Mean***	***45.19***	***11.48***	***7.72%***	***418.8***
	STD	6.49	0.97	0.12%	12.1
	Max.	29.22	9.65	7.52%	404.3
	Min.	50.38	13.32	7.82%	435.4
BSB	***Mean***	***55.23***	***12.45***	***7.10%***	***621.6***
	STD	5.81	1.16	0.07%	16.4
	Max.	46.29	11.09	7.01%	600.1
	Min.	63.48	14.81	7.19%	643.7
PPP	***Mean***	***78.72***	***14.07***	***6.67%***	***496.0***
	STD	11.84	0.76	0.17%	12.1
	Max.	50.24	12.75	6.50%	479.4
	Min.	93.29	14.90	6.92%	512.0

Table S2. Experimental data for each CLT specimen

Layup	Lamella	Specimen	Class1	Class2	$f_{m,k,ave}$ (MPa)	MOR (MPa)	E_{corr} (MPa)	MC (%)	ρ (kg/m³)	Failure mode Prim.	Second.	Tert.
BBB	1	1	D60	D40	50	111,11	15,74	6,98%	723,0	RS	T	D
BBB	1	2	D60	D40	50	126,13	15,80	6,98%	723,0	T	TS	
BBB	2	1	D50	D50	50	104,45	15,28	6,97%	726,1	T	RS	TS
BBB	2	2	D50	D50	50	106,47	15,05	6,97%	726,1	T	RS	TS
BBB	3	1	D50	D50	50	88,22	16,56	6,95%	750,1	RS	D	
BBB	3	2	D50	D50	50	90,38	16,47	6,95%	750,1	D	RS	TS
BBB	4	1	D60	D50	55	130,5	17,64	6,90%	739,7	T	C	RS
BBB	4	2	D60	D50	55	112,79	17,80	6,90%	739,7	RS	TS	D
BBB	5	1	D50	D50	50	107,98	14,81	6,92%	740,8	RS	T	TS
BBB	5	2	D50	D50	50	123	15,24	6,92%	740,8	D	RS	T
BPB	1	1	D60	D50	55	100,82	16,38	6,87%	671,8	RS		
BPB	1	2	D60	D50	55	109,01	15,88	6,87%	671,8	RS	TS	
BPB	2	1	D50	D50	50	107,25	16,10	6,97%	657,8	RS		
BPB	2	2	D50	D50	50	107,04	15,95	6,97%	657,8	RS		
BPB	3	1	D50	D50	50	111,63	15,91	7,00%	639,9	RS		
BPB	3	2	D50	D50	50	104,25	14,93	7,00%	639,9	RS		
BPB	4	1	D50	D50	50	114,42	15,39	7,20%	664,4	RS		
BPB	4	2	D50	D50	50	110,61	16,40	7,20%	664,4	RS		
BPB	5	1	D50	D50	50	113,97	14,68	6,84%	650,6	RS		
BPB	5	2	D50	D50	50	108,31	15,42	6,84%	650,6	RS		
SPS	1	1	C35	C35	35	62,11	16,15	7,16%	463,1	T		
SPS	1	2	C35	C35	35	69,52	16,71	7,16%	463,1	RT		
SPS	2	1	C24	C24	24	66,02	13,46	7,48%	491,2	RT	RS	
SPS	2	2	C24	C24	24	74,84	14,63	7,48%	491,2	RT	RS	
SPS	3	1	C30	C24	27	70,94	13,74	7,37%	477,7	RT	TS	RS
SPS	3	2	C30	C24	27	51,63	11,83	7,37%	477,7	C	T	
SPS	4	1	C24	C24	24	45,6	13,42	7,46%	487,6	T		

SPS	4	2	C24	C24	24	51,45	13,62	7,46%	487,6	T		
SPS	5	1	C35	C27	31	61,9	15,90	7,30%	448,0	RT	TS	
SPS	5	2	C35	C27	31	51,12	13,18	7,30%	448,0	T		
SSS	1	1	C27	C24	25,5	50,21	11,34	7,75%	421,8	RS	RT	TS
SSS	1	2	C27	C24	25,5	41,04	11,95	7,75%	421,8	RS	D	
SSS	2	1	C35	C24	31	44,7	11,27	7,70%	404,3	RS	D	
SSS	2	2	C35	C24	31	29,22	9,65	7,70%	404,3	T	RS	
SSS	3	1	C35	C30	32,5	45,76	11,99	7,82%	424,8	D	RS	
SSS	3	2	C35	C30	32,5	43,34	11,04	7,82%	424,8	T		
SSS	4	1	C30	C24	27	47,44	10,98	7,82%	407,6	RS	D	
SSS	4	2	C30	C24	27	50,38	11,01	7,82%	407,6	RS	D	
SSS	5	1	C30	C27	28,5	49,46	13,32	7,52%	435,4	RS	D	
SSS	5	2	C30	C27	28,5	50,37	12,25	7,52%	435,4	RS	D	
BSB	1	1	D50	D40	45	63,48	11,58	7,02%	600,1	RS	D	
BSB	1	2	D50	D40	45	54,59	11,09	7,02%	600,1	D	RS	
BSB	2	1	D50	D40	45	59,24	12,39	7,01%	615,3	?		
BSB	2	2	D50	D40	45	59,77	12,62	7,01%	615,3	D	RS	
BSB	3	1	D60	D50	55	46,6	12,33	7,15%	614,4	D	RS	
BSB	3	2	D60	D50	55	54,53	12,03	7,15%	614,4	D	RS	
BSB	4	1	D60	D60	60	58,71	14,04	7,19%	643,7	D	RS	
BSB	4	2	D60	D60	60	58,49	14,81	7,19%	643,7	D	RS	
BSB	5	1	D50	D50	50	50,62	11,45	7,11%	634,2	T		
BSB	5	2	D50	D50	50	46,29	12,13	7,11%	634,2	D	RS	
PPP	1	1	C24	C22	23	50,24	13,20	6,52%	512,0	RS	RT	
PPP	1	2	C24	C22	23	88,69	14,27	6,52%	512,0	RS	T	
PPP	2	1	C24	C24	24	80,79	14,59	6,50%	500,3	RS		
PPP	2	2	C24	C24	24	86,64	13,34	6,50%	500,3	RS	T	
PPP	3	1	C27	C22	25,5	75,75	14,43	6,92%	487,0	T	RS	
PPP	3	2	C27	C22	25,5	81,75	13,71	6,92%	487,0	C	T	
PPP	4	1	C27	C24	25,5	77,59	14,65	6,76%	479,4	RS		
PPP	4	2	C27	C24	25,5	80,72	14,81	6,76%	479,4	RS		

PPP	5	1	C27	C24	25,5	93,29	14,90	6,62%	501,1	RS		
PPP	5	2	C27	C24	25,5	71,74	12,75	6,62%	501,1	T	RS	D

Note: each panel consisted of 5 pairs of face layer lamellae, and 2 specimens were cut from each pair.

Legend:

- Class 1 and Class 2 are the top and bottom layer lamella strength classes, respectively
- $f_{m,k,ave}$ – average face layer characteristic bending stress
- MOR, E_{corr}, MC and ρ are bending strength, corrected Modulus of Elasticity, Moisture Content and Density, respectively
- Failure modes: C = Compression, D = Delamination, RS = Rolling Shear, RT = Brittle Tensile, T = Tensile, TS = Top Layer Shear
  Specimens often exhibited a combination of several failure modes; primary, secondary and tertiary modes are indicated.
